# The immune-related role of beta-2-microglobulin in melanoma

**DOI:** 10.3389/fonc.2022.944722

**Published:** 2022-08-16

**Authors:** Chuqiao Wang, Zeqi Wang, Tengteng Yao, Jibo Zhou, Zhaoyang Wang

**Affiliations:** ^1^ Department of Ophthalmology, Shanghai Ninth People’s Hospital, Shanghai Jiao Tong University School of Medicine, Shanghai, China; ^2^ Shanghai Key Laboratory of Orbital Diseases and Ophthalmic Tumor, Shanghai Ninth People’s Hospital, Shanghai Jiao Tong University School of Medicine, Shanghai, China; ^3^ Department of Ophthalmology, Shanghai Tenth People’s Hospital Affiliated to Tongji University, Shanghai, China

**Keywords:** Beta-2-microglobulin (β2M), major histocompatibility complex (MHC) class I, T cells, melanoma, immunotherapy

## Abstract

Despite the remarkable success of immunotherapy in the treatment of melanoma, resistance to these agents still affects patient prognosis and response to therapies. Beta-2-microglobulin (β2M), an important subunit of major histocompatibility complex (MHC) class I, has important biological functions and roles in tumor immunity. In recent years, increasing studies have shown that B2M gene deficiency can inhibit MHC class I antigen presentation and lead to cancer immune evasion by affecting β2M expression. Based on this, B2M gene defect and T cell-based immunotherapy can interact to affect the efficacy of melanoma treatment. Taking into account the many recent advances in B2M-related melanoma immunity, here we discuss the immune function of the B2M gene in tumors, its common genetic alteration in melanoma, and its impact on and related improvements in melanoma immunotherapy. Our comprehensive review of β2M biology and its role in tumor immunotherapy contributes to understanding the potential of B2M gene as a promising melanoma therapeutic target.

## 1 Introduction

Melanoma is the most aggressive and dangerous form of cancer that develops from transformed melanocytes. Melanoma is widely considered to be one of the most immunogenic cancers due to its high genomic mutational load and expression of cancer-specific or related antigens, so it has a strong potential to elicit recognition by anti-tumor CD8+ T cells and is sensitive to immunotherapy (1). Over the years, a variety of immunotherapies including immune checkpoint blockade (ICB) and adoptive cell therapy (ACT) have been developed with impressive results in the treatment of melanoma. Although these immunotherapies effectively prolong the median survival of this aggressive cancer and greatly improve the management of the disease, unfortunately, more than half of melanoma patients treated with immunotherapy have some degree of intrinsic resistance or develop acquired resistance to the treatment (2–4). And immune-related adverse events also complicate the treatment. A deeper understanding of effective biomarkers associated with immunotherapy efficacy and mechanisms of immunotherapy resistance is still needed to enable earlier detection of progressive disease and develop novel therapeutics that improve efficacy.

**Figure 1:**
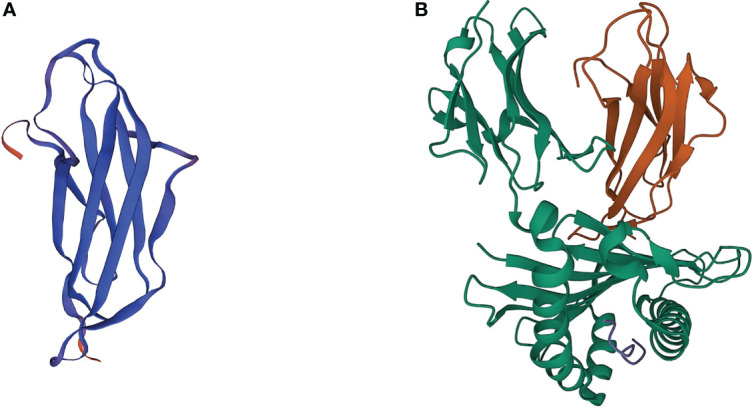
3D crystal structures of β2M and MHC class I complex. **(A)** 3D crystal structures of human β2M. **(B)** 3D crystal structures of β2M and HLA-B complex (PDB: 3BP4). Orange ribbons indicate β2M, while green ribbons indicate the HLA proteins.

The human beta-2-microglobulin (β2M) gene, B2M, is a small gene located on chromosome 15 (15q21.1), consisting of 4 exons that encode a full-length nonglycosylated protein (12k Da) composed of 119 amino acids. The B2M gene sequence shares a certain homology with genes of the immunoglobulin (Ig) constant region and the α3 domain of the major histocompatibility complex (MHC) class I molecule, so the β2M, which is encoded by B2M, and α3 domain display a similar structure as the Ig fragment c (Ig Fc) CH3 domain seven-stranded β-sheet (**Figure 1**). The β2M is synthesized by most nucleated cells and exists in two forms, membrane and free soluble β2M. Accumulating data suggests that the free β2M is one of the most important prognostic factors and predictors of survival for various types of cancers. A study in metastatic melanoma reveals that serum levels of β2M are elevated in 24% of patients before treatment, and changes in serum levels of β2M show a good correlation with disease progression (5). The membrane β2M, as a small invariable light chain, is noncovalently associated with the heavy chain of the MHC class I molecule, also known as human leukocyte antigen (HLA), on cell surfaces (6). Because it is not anchored to the cell membrane, the β2M shedding from cell surfaces or released intracellularly can exchange with free β2M present in body fluids. β2M is extensively involved in various physiological and pathological functions in tumor cells, such as cell proliferation, migration, apoptosis, and metastasis (7). However, its best-characterized function is to participate in the formation of MHC class I complexes, which play a fundamental role in tumor immunoregulation by presenting tumor antigens to activate CD8^+^ cytolytic T lymphocytes (CTLs) and regulating the cytolytic activity of natural killer (NK) cells (8, 9). Recent studies have proposed several genetic drivers of innate or acquired resistance to ICB therapy, some of which confirm that the B2M gene impedes the success of cancer immunotherapy by generating MHC class I-loss tumor variants.

This paper is a literature review focused on the role of B2M gene in tumor immunity in melanoma, in which B2M gene mutations are the common mechanism for the total loss of MHC class I antigen expression. Here we describe how β2M is involved in MHC class I-restricted tumor antigen presentation, different genetic mechanisms of B2M alterations in melanoma tissues and cell lines, and the impact of β2M deficiency on anti-melanoid immune responses and immunotherapy. And We also mention some feasible and promising approaches to improve resistance to immunotherapy caused by B2M defects.

## 2 β2M as a component of MHC class I participates in tumor antigen presentation

**Figure 2:**
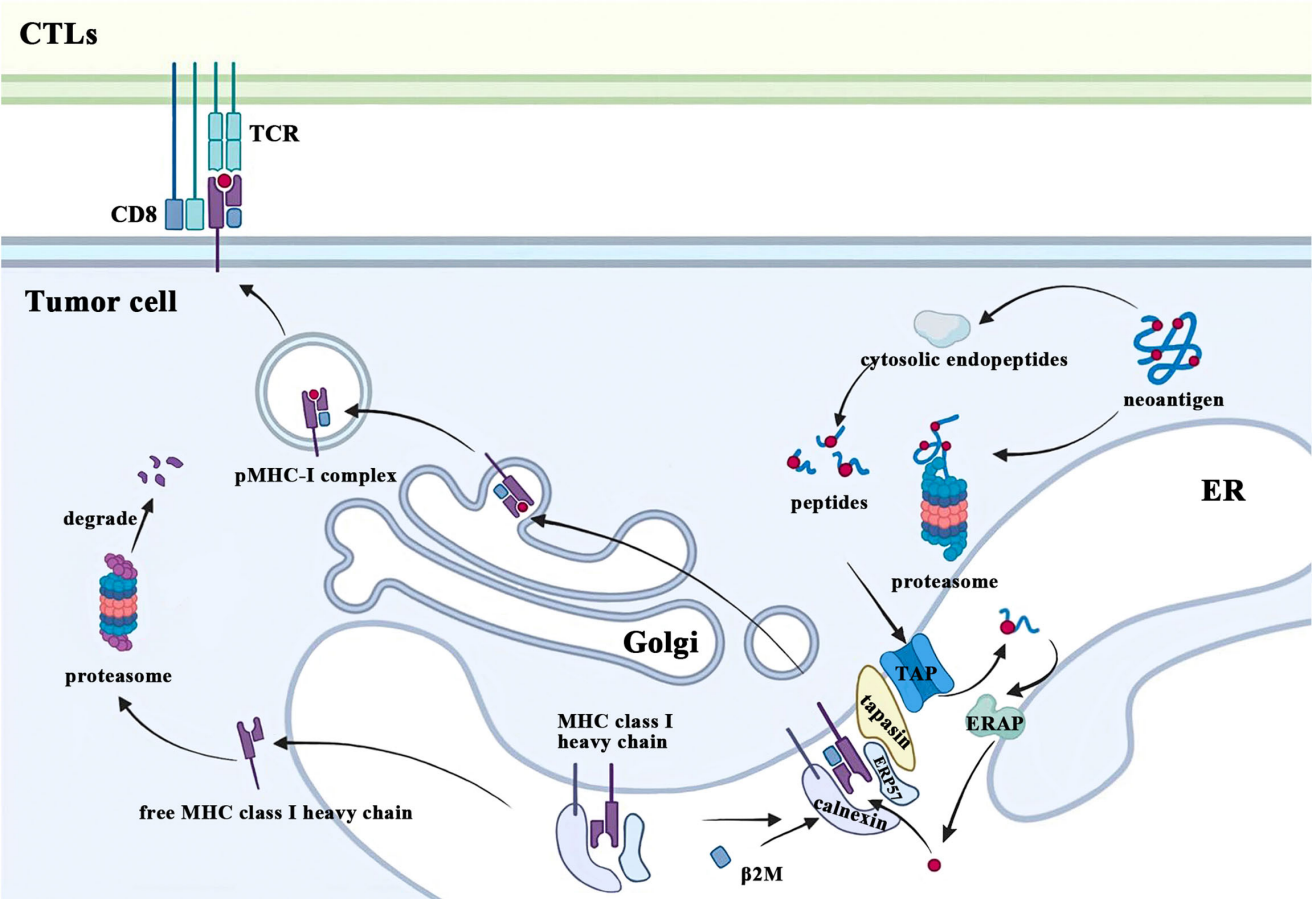
β2M is involved in the process of MHC class I antigen presentation. CTLs, cytolytic T lymphocytes; TCR, T cell receptors; ER, endoplasmic reticulum; β2M, beta-2-microglobulin; MHC, major histocompatibility complex; pMHC-I, peptide-MHC class I; TAP, transporter associated with antigen processing; ERAP: endoplasmic reticulum aminopeptidases; ERP57, endoplasmic reticulum resident protein 57.

Tumor immune surveillance requires CD8^+^ T cells to recognize and eliminate tumor cells bearing MHC class I molecules with novel peptides due to accumulated cellular stress and mutations during tumorigenesis. One of the most fundamental and important functions of β2M is to participate in this MHC class I-restricted tumor antigen presentation machinery (APM), which includes four main steps: 1) protein breakdown; 2) peptide transport and trimming; 3) assembly of MHC class I complex; 4) antigen presentation (**Figure 2**). Ubiquitinated proteins in the cytoplasm of malignant cells are first transported to the proteasome for deubiquitination and degradation, followed by being released into the cytoplasm again (10). These degradation products are actively transported to the endoplasmic reticulum (ER) by the transporter associated with antigen processing (TAP) and further trimmed into small peptides of 8 to 9 amino acids in length for MHC class I stable binding (11, 12). After cotranslationally inserting into the ER under the coordinated action of multiple chaperone proteins, free MHC class I heavy chain and β2M recognize and bind these modified peptides. Subsequently, the peptide-MHC class I (pMHC-I) complexes translocate from the ER to the Golgi and finally migrate to the plasma membrane where they can be recognized by and interact with the T cell receptors (TCR) on CTLs, which play a crucial role in eradicating tumor cells. The conformational change of the heavy chain due to β2M binding alters its interaction with chaperones such as calreticulin and TAP (13, 14). In addition, the β2M subunit is also necessary for the proper folding of the MHC class I heavy chain. After the cotranslational translocation of MHC class I heavy chains into the ER, two disulfide bonds are formed respectively within its α2 and α3 domains (15). β2M not only provides another disulfide bond for classical Ig folds but also facilitates intrachain disulfide bond formation in MHC class I molecule heavy chains, which promotes conformational changes of heavy chains and enables it to form stable trimolecular complexes with β2M and peptides (15, 16). In contrast, β2M-free MHC class I heavy chains would be retrotranslocated from the ER to the cytosol and eventually degraded by the proteasome due to ER quality control (17).

## 3 The molecular mechanisms of B2M defects in melanoma

Altered surface expression of MHC class I molecules has been frequently observed in a high proportion of various types of malignancy, among which the proportion is about 67% in melanoma and even higher in metastatic melanoma (18, 19). The heterogeneous molecular mechanisms that cause this defect can be divided into two groups, reversible and irreversible, based on whether MHC class I expression can be recovered or upregulated after cytokine treatment or other immunotherapies (20). Reversible MHC class I alterations result from defective regulation of genes encoding the heavy chain, β2M, and antigen processing machinery components, while B2M mutations often result in irreversible MHC defects. This means that MHC class I levels in MHC class I-deficient tumor cells caused by the B2M gene alteration will not be restored regardless of the type of immunotherapy. Seven major altered MHC class I phenotypes have been defined in different tumor tissues (21, 22), of which phenotype I (total loss of MHC class I antigen expression) is frequently observed in malignant melanoma cell lines and tissue samples. Tumor cells with this phenotype do not express any MHC class I molecule on their surface, so they can escape immunosurveillance and display a higher *in vivo* tumorigenicity, proliferation rate, and migratory and invasive potential (23). Many molecular mechanisms can lead to complete loss of MHC class I antigen expression, while alterations in the B2M gene have been demonstrated to be the major cause in melanoma (24, 25). In humans, the heavy chains of MHC class I are encoded by MHC class I genes, HLA-A, HLA-B, and HLA-C, which means one HLA mutation will affect a specific MHC class I molecule. Since β2M is present in all MHC class I complexes, B2M defects would broadly impact all variants of MHC class I molecules originating from different HLA alleles. And this may be the reason why the B2M mutation may lead to a decrease in the total amount of MHC class I antigens presenting on the surface of tumor cells (26). A study involving 7630 tumors across 23 major cancer types showed that the incidence of B2M alterations was 2.0% (0.9% gene deletions, 0.6% damaging mutations, and 0.1% putative damaging mutations), including 4.5% in cutaneous melanomas (27). Another study examined β2M expression in 23 primary samples of conjunctival melanoma (CM), a rare type of mucosal melanoma, by immunofluorescence staining and revealed that three cases (14%) showed no staining of β2M while 11 cases (50%) showed weak β2M expression (28). B2M loss is the initial genetic alteration in the development of MHC class I loss, as the structural alteration in the B2M gene can be detected even in HLA-positive melanoma cells (29, 30). The immunogenicity of these HLA-positive B2M gene-altered melanomas is still reduced to varying degrees, which means reduced T cell infiltration and decreased T cell–stimulatory capacity, and this confers an advantage for dissemination and expansion of those melanoma clones with irreversible genetic defects (29). And the accumulation of B2M alterations during metastasis results in a gradual decrease to total loss of HLA class I expression (29). A variety of genetic defects in B2M were observed in tumor biopsies of 29.4% of patients with metastatic melanoma which is higher than primary melanoma (31).

There are a great variety of mechanisms of B2M alterations, and the causative mutations are not only found in various types of tumors but also in systemic amyloidosis, in which misfolded proteins caused by the B2M gene mutations exhibit a strongly enhanced propensity for amyloid aggregation (32). **Table 1** summarizes the relevant mechanisms that have been identified in melanoma, including single nucleotide substitutions, small frameshifting deletions/insertions, and loss of a large segment of chromosome 15q21. Analysis of the molecular mechanisms that contributed to the loss of functional β2M in different melanoma cell lines revealed that there is a high frequency of microdeletions/insertions in the repeat region of exon 1 and exon 2 of the B2M gene (**Table 1**). Although B2M mutations are highly frequent in high-frequency microsatellite instability (MSI-H) tumors, melanoma shows a striking similarity to the mutational patterns found in MSI-H colorectal cancers (CRCs). As in MSI-H CRC, the 8-bp CT repeat region of exon I of the B2M gene has also been described as a mutational hotspot in melanoma (24, 38). Only a small fraction of primary melanomas exhibited an MSI-H pattern and Bernal et al. did not find genomic instability during MSI phenotyping of five melanoma cell lines mutated in this region (44, 45), suggesting that mutations in this hotspot gene in melanoma may not be associated with defects in the DNA mismatch repair (MMR) system. However, Sade-Feldman et al. found somatic mutations in MMR (MSH2) were found in post-Tx and post-Tx-II samples of PatT33 (31). Microdeletion/insertions and single nucleotide substitutions often lead to a frameshift and a premature stop codon, resulting in the production of nonfunctional truncated β2M (**Table 1**). And a missense mutation causing a Cys to Trp change in the melanoma cell line VMM5b (Me15) blocks the formation of a disulfide bond in the β2M, resulting in its degradation by the proteasome (39). Somatic mutations to B2M also significantly increase the mutation burden of tumor patients (26). Since early mutational events are present in a larger proportion of cancer cells, a better understanding of the timeline of mutational occurrence can be obtained by comparing the percentage of mutated alleles in the same tumor (26). High percentiles of B2M mutations found in the study suggest that B2M mutations may occur early in tumor development (26). It is also worth noting that the coexistence of two different B2M gene mutations has not been detected in all studies involving genetic mechanisms of B2M-deficient melanoma cell lines, suggesting the allelic B2M loss. Detection of chromosome 15 by microsatellite markers or single nucleotide polymorphism (SNP) array analysis found loss of heterozygosity (LOH) in almost all B2M-deficient melanoma samples (45). Therefore, the loss of B2M expression in melanoma is typically caused by the coincidence of mutational events involving both copies of the B2M gene: a mutation in one B2M allele and the loss of the second B2M allele. In fact, the same partial deletion of chromosome 15q as in B2M-deficient tumor cells was also found in some melanoma cell lines without B2M mutations (29). This suggests that the chronological sequence of genetic alterations in B2M defects is as follows: a large deletion encompassing the B2M gene was first acquired on one chromosome 15q, followed by differentiation and independent acquisition of an additional small mutation affecting the second B2M allele. Thus, B2M allele loss owing to chromosome 15 instability is a more frequent form of B2M alteration, and its frequency in melanoma metastases is higher than that of B2M gene mutation. One study showed that LOH in the B2M region on chromosome 15q21 was detected in 16% of metastatic melanoma samples (n = 70) (46). At the same time, since β2M and MHC class I molecules can normally express in melanoma cells with only LOH on chromosome 15q, such chromosomal structural changes may be overlooked. It is also worth highlighting that loss of MHC class I expression was detected in all melanoma cell lines with B2M mutations in current published research (mentioned in **Table 1**), whereas in other tumor cell lines with B2M mutations, the expression of MHC class I may not necessarily be detected simultaneously. For example, in many colon cancer cell lines, mutations in B2M lead to the synthesis of inefficient β2M variants that only reduce the number of MHC class I molecules on the tumor cell surface (47–49). Besides these irreversible defects mentioned above, other mechanisms can also affect B2M expression, e.g., epigenetic regulation of B2M transcription. The data from The Cancer Genome Atlas (TCGA) indicated that the methylation of the NLRC5 promoter was negatively correlated with the expression of β2M in melanoma, resulting in reduced expression of MHC class I and anti-tumor immune evasion (50).

**Table 1:** Summary of B2M mutations in melanoma.

Cell line/Tumor tissue	The origin of tumor	B2M gene mutation	B2M mRNA	β2M	Chromosome 15 aberrations	Ref
FO-1		(1) a deletion of Exon I				(33)
		(2)a deletion of a segment of Intron I				
SK-MEL-33	a metastatic melanoma involving regional lymph nodes in the left axilla	a guanosine deletion in codon 76 in Exon II	a frameshift and premature termination	a truncated protein (18 amino acids shorter)		(34)
Me1386	a metastatic lesion	a CT deletion in the 8-bp CT repeat region of Exon I	(1)a shift in the reading frame starting at nucleotide position 45		abnormalities in chromosome 15	(24)
			(2)a premature UGA stop codon at nucleotide position 165			
Me9923/Me9923P	a metastatic/primary lesion	(1) a 14-bp deletion in Exon II		a putative COOH-terminal truncated protein of 83 amino acids	abnormalities in chromosome 15	
		(2) a C→G transversion mutation at nucleotide position 258	an in-frame premature stop at codon 86			
Me18105	a metastatic lesion	a point mutation (A→G) at the splice acceptor site of Intron I	a 11-bp deletion creating a frameshift and premature termination		abnormalities in chromosome 15	
LB1622-MEL	a in-transit metastatic lesion	a point mutation (T→A) in the start codon of Exon I			a partial deletion on chromosome 15q (15q21-15q22)	(35)
BB74-MEL	a adrenal gland metastasis	a point mutation (C→G) at codon 31 in Exon II			a partial deletion on chromosome 15q22	
GR34	a primary lesion	a deletion of 4 bases (TTCT) in the 8-bp CT repeat region of codons 15–16 in Exon I	the appearance of a stop codon at position 42	a truncated non-functional 41 amino acid β2M	a partial deletion on chromosome 15q	(36)
UKRV-Mel-2b	a pleural effusion 8 months after diagnosis	a 498-bp deletion including the whole Exon I			an extensive deletion of sequences from one chromosome 15q	(37)
1074MEL	a recurrent metastatic lesion	a point mutation (G→A) at the translation initiation codon	abolish the initiation of translation		LOH in chromosome 15	(38)
1174MEL	a recurrent metastatic lesion	a point mutation (C→G) at codon 31	the introduction of a premature TGA stop codon		LOH in chromosome 15	
1106MEL	a recurrent metastatic lesion	a CT deletion in the 8-bp CT repeat region of codons 13–15 in Exon I			LOH in chromosome 15	
1180MEL	a recurrent metastatic lesion	a CT deletion in the 8-bp CT repeat region of codons 13–15 in Exon I			LOH in chromosome 15	
1259MEL	a recurrent metastatic lesion	a CT deletion in the 8-bp CT repeat region of codons 13–15 in Exon I			LOH in chromosome 15	
VMM5B	a metastatic lesion	a point mutation (C→G) at codon 45 in Exon II		misfolding and degradation of β2M	LOH in chromosome 15	(39)
Mel249	a metastatic lesion	a 2-bp microdeletion in codon 62 in Exon II			broad-range deletions within chromosome 15	(40)
Mel499	a metastatic lesion	a point mutation (T→ A) at position two of the Intron I which destroys the GU donor splice consensus site of Intron I	the insertion of intron I sequences of different lengths, 27 and 407 bp		LOH in chromosome 15	
Mel505	a metastatic lesion				broad-range deletions within chromosome 15	(40)
Mel592	a metastatic lesion				broad-range deletions within chromosome 15	
DNR-DC-M010	a right inguinal lymph node metastasis	a point mutation (G→ T) at codon 67 in Exon II	an early stop codon	a short version of β2M	LOH in chromosome 15	(30)
Ma-Mel-48c	lymph node metastases one year after diagnosis of stage IV disease	a 60-bp deletion starting at codon 96 in Exon II			a partial deletion on chromosome 15q (15q13.3-15q21.2)	(29)
Ma-Mel-100b	regional lymph node metastases three years after diagnosis of stage III disease	a 12-bp deletion in Exon II			a partial deletion on chromosome 15q (15q12-15q22.2)	
Ma-Mel-86b	a late recurrent lymph node lesion 1.5 years after diagnosis of stage IV disease	the B2M gene and flanking sequences were lost			a large deletion on chromosome 15q (15q11.2-15q22.31)	(41)
Ma-Mel-86f	a late recurrent lymph node lesion 6 years after diagnosis of stage IV disease	the deletion affected only the B2M gene			a large deletion on chromosome 15q (15q11.2-15q22.31)	(41)
M437	a metastatic lesion	a 4-bp S14 frameshift deletion in Exon I				(42)
Pat208	a metastatic lesion	frameshift mutations in Exon I: p.Leu13fs and p.Ser14fs			LOH in chromosome 15	(31)
PatT33	a metastatic lesion	two B2M frameshift mutations: p.Ser14fs and p.Gly63fs			no LOH in chromosome 15	
	a lymph node metastasis	a 28-bp deletion	a frameshift and truncation starting from codon 3			(43)

## 4 Effect of B2M defects on anti-melanoma immune responses

Compelling Evidence shows that β2M deficiency affects tumor immunity by hampering MHC class I-mediated tumor antigen presentation in tumor lesions and contributes to a poor clinical outcome in melanoma. The high intrinsic immunogenicity of melanoma cells makes CTLs recognize multiple peptide epitopes from different types of tumor antigens presented by MHC class I (51, 52), whereas β2M can participate in the presentation of all MHC class I antigens. As a consequence of the poor tissue availability of consecutive melanoma metastases, related melanoma cell lines, and autologous peripheral blood T lymphocytes, only a few studies have kept track of the evolution of melanoma immunogenicity during disease progression (29, 53, 54). However, studies of metastatic cell lines and tissue samples have identified the complete lack of HLA class I antigen expression due to B2M gene deficiency as one of several genetic alterations affecting the tumor cells’ immunogenicity. As shown in a study that monitoring three consecutive melanoma metastases from a patient with melanoma, as the tumor progressed, accumulated alterations in the B2M gene acquired by melanoma cells gradually decreased the immunogenicity of tumor cells, which culminated in impaired T-cell recognition caused by the irreversible total lack of HLA class I antigen expression (29). At the same time, the activated CTLs release large amounts of cytokines, such as interferon-γ (IFN-γ) and tumor necrosis factor-α (TNF-α), leading therefore to the formation of the tumor microenvironment and subsequent recruitment of T cells. IFN-γ signaling would upregulate the synthesis and expression of the MHC class I, inducing a positive feedback loop that results in additional T cell recruitment and activation (55). Melanoma cell lines with B2M mutation may lose IFN-γ-mediated MHC class I inducibility, thereby supporting tumor evasion of immune surveillance (56). After transfection of wild-type B2M gene, melanoma cells with B2M defects restored sensitivity to T cell lysis and ability to induce T cell IFN-γ secretion in an HLA-restricted manner.

In addition, the role of immunity in developing tumors is twofold, not only protecting the host from tumor formation but also shaping tumor immunogenicity by promoting the outgrowth of tumor cells that have developed immune resistance mechanisms (57). During tumor development, rare tumor cells surviving after the elimination phase enter an equilibrium phase, in which the adaptive immune system blocks tumor cells’ outgrowth but also exerts selective pressure on them. Among the several immune effector mechanisms in this cancer immunoediting process, the constant immune pressure exerted by tumor antigen-specific T cells is suggested as a potent driver to promote the selection of tumor cell clones. This hypothesis implies that when B2M-defective tumor variants appear, these poorly immunogenic tumor clones become “invisible” to CTLs and thus acquire a growth advantage to take over the other clonal tumor populations (29, 38). These tumor cells that have acquired the ability to circumvent immune recognition develop resistance to the anti-tumor immune response, which leads to tumor immune escape.

MHC class I–negative tumor cells could still be targeted by innate CD56+ NK cells that recognize tumor cells through molecular mechanisms distinct from CTLs and could be another source of IFN-γ. MHC class I molecules, as ligands for inhibitory NK-cell receptors, are involved in the “licensing” or “education” of NK cells (58). The failure of surface MHC class I expression could upregulate the expression of Caspase 3 and KIR2DL1 in tumor cells to recruit NK cells and induce apoptosis. To explore whether B2M-deficient melanomas could be targeted with NK cells, a study transplanted a mixture of parental B2M+/+ and B2M-/- cell lines (in a 1:1 ratio) into mice with or without NK cells (31). The study found that in mice lacking NK cells, the number of B2M-depleted melanoma cells was significantly increased compared with B2M +/+ cells. The expression of activating NK ligands, including MHC class I-related chain A/B (MICA/B) and UL16 binding protein (ULBP), was positive on B2M alteration-induced MHC class I-deficient melanoma cells (30), and the coculture of these cells with NK cells found that they were efficiently killed by NK cells compared with MHC class I-positive melanoma cells (40). Nevertheless, studies detected a low tumor infiltration of CD56+ NK cells in melanoma metastases regardless of the HLA expression (30, 41). Therefore, NK cells could kill these metastatic melanoma cells but rarely migrate into the melanoma lesions due to their potentially impaired migratory function *in vivo (* 30). Moreover, the imbalance between activating and inhibitory signals decreases NK cell cytotoxicity against malignant cells, supported by a less robust activity of tissue-infiltrating NK cells in solid tumors and low levels of MICA and ULBP expression in some B2M-deficient melanoma cell lines (59).

## 5 Association between B2M genetic alterations and immunotherapy

Cancer immunotherapies that enhance the ability of the immune system to eliminate malignant cells and overcome immune escape are now being recognized as the potentially most promising therapies for various cancers. Melanoma is also among the most sensitive malignancies to immune modulation. B2M mutations are closely associated with patient progression on immunotherapy in melanoma. Loss of the B2M gene may lead to innate and acquired resistance to immunotherapy methods on T cell immunity, which means an intrinsic lack of response to the initial therapy, or subsequent progression or new metastases to the site of metastases after the initial response. Here we summarize resistance to different immunotherapeutic approaches caused by B2M disruption in melanoma and promising treatment strategies to overcome it.

### 5.1 Immune checkpoint blockade therapy

#### 5.1.1 B2M alterations induce ICI resistance while ICIs promote B2M mutations

Immune checkpoint blockade (ICB) therapy is designed to target immune checkpoint receptors on tumors and immune cells, such as programmed cell death-1 (PD-1), programmed death ligand-1 (PD-L1) or cytotoxic associated lymphocyte antigen-4 (CTLA-4), to restore CD8^+^ T cell-induced anti-tumor immune activity (2, 3, 60). Despite the significant improvements seen in melanoma prognosis with intensive studies on immune checkpoint inhibitors (ICIs), a substantial fraction of patients nevertheless suffers from relapsed disease and eventually die due to treatment resistance. Because the efficiency of ICIs depends on CTLs’ recognition of MIHC class I antigens presenting tumor antigen-derived peptides, β2M, as a key factor required for the assembly of MHC class I complexes and the stable presentation of tumor antigens, its deficiency may be a common genetic mechanism of resistance to ICB therapy. Not only that, but strong and persistent T cell selection pressure generated by ICIs also leads to the preferential selection of B2M gene mutations. After an early analysis of molecules involved in functional HLA class I Ag expression in melanoma cell lines originating from recurrent metastases after initial T cell-based immunotherapy, three types of B2M gene mutations resulting in a lack of translation of the β2M were identified (38). Support from correlative clinical samples has been lacking for a long time until Zaretsky et al. identified homozygous B2M truncating mutations in the baseline and progressive lesions of a patient with pembrolizumab-resistant advanced melanoma (42). A similar alteration in B2M with corresponding LOH was also found in a melanoma patient who had a partial response to nivolumab (61). Rodig et al. assessed the expression of MHC class I and II proteins on tumor cells from 181 patients with melanoma before ICB therapy and correlated the results with clinical outcomes of different ICIs treatments and transcriptomic and genomic profiling in selected cases (62). Loss of MHC class I expression in the majority (>50% of cells) of melanoma cells was observed in 78 of these patients and associated with transcriptional downregulation of HLA and B2M genes. However, they also concluded that this loss of MHC class I expression predicted primary resistance to anti-CTLA-4 therapy, but not anti-PD-1 therapy, which seems inconsistent with the report by Zaretsky et al. Defects in the B2M gene may be an effective predictive marker for ICB therapy in melanoma. In a cohort of metastatic melanoma patients treated with several ICB therapies, Moshe et al. identified B2M aberrations in 29.4% of patients with progressing disease, including point mutations, deletions, or LOH (31). They also found in two independent cohorts of melanoma patients treated with different ICIs that B2M LOH events were significantly enriched in non-responders (about three times as many as responders) and associated with worse overall survival. In contrast, results from a larger cohort of 131 patients with metastatic melanoma showed high levels of β2M expressed either in tumor or stroma are associated with a good response to immunotherapy (63). Uveal melanoma (UM) is a rare subset of melanoma (3%-5% of all melanomas) but also the most common intraocular tumor in adults. UM shares a common origin with cutaneous melanoma, but ICB therapy has had limited success in the clinical treatment of UM due to their different mutational load and antigen expression. Interestingly, there was no difference in B2M expression observed between cutaneous melanoma and UM metastases despite different responses to ICIs (64).

#### 5.1.2 Combination therapy in overcoming ICI resistance with B2M mutation

Talminogene lapherparevec (TVEC) is an FDA-approved genetically-modified herpes simplex virus for the treatment of advanced melanoma (65). Khaddour et al. reported a case of a patient with rapidly progressive immunotherapy refractory intracranial metastatic melanoma who had acquired B2M mutation after initial ICB therapy (66). The patient had a durable complete response to the visceral and intracranial metastatic disease after the therapy of sequential TVEC and pembrolizumab followed by temozolomide. The researchers proposed that local injection of oncolytic viruses may activate the type I IFN pathway, which may explain the reason why TVEC can avoid ICB resistance due to B2M mutations and enhance anticancer immune responses.

In addition, cytokines, as powerful modulators of the immune system, can significantly enhance the immune response to tumors. Bempegaldesleukin, also known as NKTR-214, is a prodrug of conjugated IL2 that provides sustained activation of the IL-2 pathway and leads to a systemic expansion of both CD4+ and CD8+ T cells (67). Combination therapy with bempegaldesleukin and ICI revealed a synergistic antitumor effect, and the systemic administration of bempegaldesleukin attenuated anti-PD-1 resistance in B2M knockout tumors and prolonged survival in B2M knockout melanoma mice (68, 69).

### 5.2 Cancer vaccines

Cancer vaccines are designed based on specific tumor antigens to induce or enhance anti-tumor immune responses in cancer patients in a preventive or therapeutic way. However, T-cell-based cancer vaccines may develop resistance due to the deficiency of B2M. As early as 1998, Benitez et al. reported B2M mutations identified in two metastatic melanoma patients with tumor progression after immunizations with MAGE peptides (35). In one case report, investigators tracked the chronological sequence of appearance of B2M gene mutation in several successive metastatic melanoma lesions and a lymph node biopsy-derived cell line obtained after immunotherapy with a dendritic cell vaccine (DCV) transfected with autologous tumor mRNA (30). Although this therapy stimulates T cells by the presentation of tumor antigens by DC, helping to eliminate the effects of the loss of MHC class I surface expression in tumor cells, B2M gene defect found in this patient with aggressive clinical course and resistance to DC vaccination might partly explain the failure of the therapy. Subsequently in a study of the application of an RNA-based poly-neoepitope individualized mutanome vaccines in melanoma patients, Sahin et al. reported that one of these patients had a late relapse due to the outgrowth of B2M-deficient melanoma cells after receiving the vaccine (70). In a randomized phase II trial, by analysis of blood samples from melanoma patients treated with two vaccines featuring autologous tumor antigens respectively, researchers found that β2M expression was negatively correlated with immune response and survival in patient-specific autologous DCVs, but positively correlated with those in autologous tumor cell vaccines (TCV) (71).

### 5.3 B2M gene delivery

Many *in vitro* experiments have shown that using the plasmids or adenoviruses strategy to deliver wild-type human B2M gene into melanoma cells or other tumor cells can restore tumor cell HLA class I antigen expression and peptide-specific T lymphocyte recognition (24, 29, 30, 37, 72). An *in vivo* study using the Ma-Mel-86b tumor xenograft model in nude mice also showed that the intratumoral injection of B2M-carrying vectors resulted in restoring regular HLA class I expression (72). This mechanism is one of the bases of the Allovectin-7 design. Allovectin-7 consists of a bicistronic plasmid DNA (VCL-1005) encoding two transgene proteins, HLA-B7 and β2M (73, 74). In transfected melanoma cells, β2M can not only combine with HLA-B7 encoded on plasmids but also combine with other endogenous MHC class I heavy chains, thereby enhancing the expression of multiple MHC class I molecules on the cell surface (75). An *in vitro* study indicated that compared with VCL-1004, a plasmid encoding only HLA-B7, cell lines lacking/not lacking endogenous β2M expression transfected with VCL-1005 had significantly increased surface MHC class I expression (75). The phase I clinical trial demonstrated the intratumoral administration of Allovectin-7 delivers these two genes into tumor cells, enabling the synthesis and expression of intact MHC class I complexes on the tumor cell surface and stimulating T cell-based immune responses to transfected cells and foreign antigens (76–78). One Phase II clinical trial evaluated the safety and efficacy of Allovectin-7 among 133 patients (127 evaluable for efficacy) with stage III or IV metastatic melanoma (79). The overall response rate was 11.8%, including 8.7% complete response and 3.1% partial response. The median duration of response, TTP and median overall survival were 13.8, 1.6 and 18.8 months, respectively. While the Phase II results of Allovectin-7 showed a surprising therapeutic effect, the Phase III Allovectin Immunotherapy for Metastatic Melanoma (AIMM) trial found that responders to Allovectin-7 had significantly shorter overall survival (18.8 months versus 24.1 months, P = 0.491) despite a longer duration of response compared with controls (intravenous dacarbazine or oral TMZ) (80). Although the Phase III trial of Allovectin-7 failed to meet key endpoints, the success of its Phase I/Phase II trials and related basic studies still demonstrates that B2M gene delivery can address the problems its mutation poses to T-cell-based tumor immunity. We believe that the development of immunotherapy based on this mechanism will bring more treatment options to patients with immunotherapy refractory melanoma caused by mutations. It is expected that gene therapy will have a huge development space and a promising future in tumor immunotherapy.

### 5.4 Natural killer cell-based therapy

In contrast to T cells, NK cells are essential components of the innate immune system and do not need prior activation to target tumor cells. The activity of NK cells is dependent on a complex interaction of activating and inhibiting receptors on their surfaces. Experiments have shown that malignant melanoma cells express a set of ligands that mediate NK cell-activating receptor recognition, triggering NK cells to become active killers (81). Low or absent HLA class I expression leads to the lack of killer immunoglobulin-like receptors (KIR) engagement, which switches NK cells from a state of equilibrium to activation by decreasing inhibitory signal input (82). Due to the natural activity of NK cells against melanoma cells and their strong killing effect on tumor cells with reduced expression of MCH class I molecules, NK cell-based therapy is promising immunotherapy for ICI-resistant melanoma caused by β2M deficiency (83, 84). However, multiple studies have demonstrated impaired NK cell activity in melanoma patients, and immuno-suppressive tumor microenvironment and inadequate NK cell homing also negatively affect NK cell therapy (85–88). The combination of NK cell-based therapy with other therapies may synergistically improve NK cell recognition and avoid tumor immune evasion (89, 90).

### 5.5 Chimeric antigen receptor T cell therapy

Currently, adoptive cell therapy (ACT), as a new strategy for improving the treatment of metastatic melanoma, is primarily focused on patients resistant or non-tolerant to the ICIs. The specificity of tumor-associated antigens (TAA) recognition by chimeric antigen receptor (CAR) T cells is defined by the antibody domain, thus freeing antigen recognition from MHC restriction. Therefore, adoptive CAR-T cell therapy is expected to be a potential option for patients with MHC class I antigen presentation deficiency due to B2M defects. Furthermore, disruption of the B2M gene to generate MHC class I-deficient T cells *via* CRISPR/Cas9 can significantly reduce the surveillance of allogeneic T cells and may prevent host rejection driven by HLA differences (91). Although evidence from current clinical studies is very limited in CAR-T cell therapy for melanoma, it may offer new hope for patients with metastatic melanoma.

## 6 Conclusion

The exploration of potential clinical implications of the B2M gene in melanoma immunity is just starting. Complete loss of MHC class I expression caused by β2M deficiency contributes to immune selection and expansion of melanomas with such genetic defects, while also contributing to immune escape and leading to resistance to T cell-based immunotherapy. Therefore, β2M is gradually becoming a potential biomarker and therapeutic target for melanoma immunotherapy. To a certain extent, the detection of B2M mutations can predict the efficacy of T cell-based immunotherapy in melanoma. B2M gene delivery also provides a new solution to the problem of immune resistance caused by gene mutations. The development and clinical validation of different delivery vehicles such as viruses, plasmids, and nanomaterials are all possible directions for future studies. New technologies and hypotheses in melanoma tumor immunity are increasingly explored, which may provide additional insights into our understanding of the role of B2M in tumor immunity. Meanwhile, additional studies about new therapeutic strategies for B2M-deficient melanoma are still needed to pave the way for advanced melanoma control and eradication.

## Author contributions

CW drafted and revised the manuscript and figures. JZ and ZhW are the corresponding authors who instructed the research. ZeW and TY helped with revising and editing. All authors contributed to the artide and approved the submitted version.

## Funding

We acknowledge funding supports from the National Natural Science Foundation of China (81770934); the Program of Science and Technology Innovation Action of Science and Technology Commission of Shanghai Municipality (22Y11910100); the Scientific research project of Shanghai Municipal Health Commission (202140416); and Interdisciplinary research joint fund project of Tongji University(2022-4-ZD-09).

## Conflict of interest

The authors declare that the research was conducted in the absence of any commercial or financial relationships that could be construed as a potential conflict of interest.

## Publisher’s note

All claims expressed in this article are solely those of the authors and do not necessarily represent those of their affiliated organizations, or those of the publisher, the editors and the reviewers. Any product that may be evaluated in this article, or claim that may be made by its manufacturer, is not guaranteed or endorsed by the publisher.
